# A Candidate Regulatory Variant at the *TREM* Gene Cluster Confer Alzheimer’s Disease Risk by Modulating Both Amyloid-β Pathology and Neuronal Degeneration

**DOI:** 10.3389/fnins.2019.00742

**Published:** 2019-07-17

**Authors:** Mei-Ling Tian, Xiao-Neng Ni, Jie-Qiong Li, Chen-Chen Tan, Xi-Peng Cao, Lan Tan, for the Alzheimer’s Disease Neuroimaging Initiative

**Affiliations:** ^1^Department of Neurology, Qingdao Municipal Hospital, Qingdao University, Qingdao, China; ^2^Department of Neurology, Qingdao Municipal Hospital, Nanjing Medical University, Nanjing, China; ^3^Clinical Research Center, Qingdao Municipal Hospital, Qingdao University, Qingdao, China

**Keywords:** Alzheimer’s disease, *TREM2* gene, *TREML1*, rs9357347, genetic mechanism, cerebrospinal fluid, amyloid-β pathology, neuronal degeneration

## Abstract

**Background:** rs9357347 located at the triggering receptor expressed on myeloid cells (*TREM*) gene cluster could increase TREM2 and TREM-like transcript 1 (TREML1) brain gene expression, which is considered to play a protective role against Alzheimer’s disease (AD).

**Objectives:** To investigate the role of rs9357347 in AD pathogenesis by exploring the effects of rs9357347 on AD specific biomarkers.

**Methods:** This study analyzed the association of rs9357347 with AD-related cerebrospinal fluid (CSF) and neuroimaging markers from 201 cognitively normal (CN) older adults, 349 elders with mild cognitive impairment (MCI), and 172 elders with AD dementia from the Alzheimer’s Disease Neuroimaging Initiative (ADNI). We next analyzed the association in 259 amyloid-β positive (Aβ+) elders and 117 amyloid-β negative (Aβ-) elders (Aβ+: CSF Aβ_1-42_ ≤ 192 pg/ml; Aβ-: CSF Aβ_1-42_ > 192 pg/ml). Associations were tested using multiple linear regression models at baseline. Furthermore, multiple mixed-effects models were used in a longitudinal study which lasted 4 years.

**Results:** At baseline, we found that rs9357347 had association with CSF Aβ_1-42_ in CN group (β = 0.357, *P* = 0.009). In AD group, rs9357347 was associated with total tau (T-tau) level (β = -0.436, *P* = 0.007). Moreover, the strong influence exerted by rs9357347 on T-tau was also seen in Aβ+ group (β = -0.202, *P* = 0.036). In the longitudinal study, rs9357347 was also found to be associated with Aβ_1-42_ in CN group (β = 0.329, *P* = 0.023). In AD group, the mutation of rs9357347 was associated with slower accumulation of T-tau (β = -0.472, *P* = 0.002) and tau phosphorylated at threonine 181 [P-tau 181 (β = -0.330, *P* = 0.019)]. Furthermore, the obvious influence exerted by rs9357347 on T-tau was also seen in Aβ+ group (β = -0.241, *P* = 0.013).

**Conclusion:** This study suggested that rs9357347 reduced the risk of AD by modulating both amyloid-β pathology and neuronal degeneration.

## Introduction

Alzheimer’s disease (AD) is a complex polygenetic disease characterized by the presence of extracellular deposits of the amyloid-β_1-42_ (Aβ_1-42_) and intracellular twisted strands of the tau protein (Alzheimer’s Association, 2011). In the genetic studies, the triggering receptor expressed on myeloid cells (*TREM*) gene cluster on chromosome 6p21.11 has been identified as significantly associated gene region with AD (Karch et al., 2014). Among the *TREM* genes, a rare variant rs75932628 (encoding p. Arg47His) in *TREM2* was reported to be associated with the highest risk of developing AD in Caucasians (Colonna, 2003; Piccio et al., 2007).

Recently, Carrasquillo et al. (2017) re-analyzed whole genome and exome sequencing data to test which common AD-related variants within the *TREM* gene cluster influence AD through gene expression. rs9357347, located downstream from *TREML2* and upstream from *TREM2*, was found to be associated with AD risk (Carrasquillo et al., 2017). And the locus was demonstrated to influence *TREML1* and *TREM2* expression in the temporal cortex (Ford and McVicar, 2009). Meanwhile, they found that only *TREM2* and *TREML1* had reliable expression in the brain region. Thus, they point out that rs9357347^CC^ exerts protective effects on AD through increasing *TREML1* and *TREM2* brain expression levels. However, the specific pathogenic effect rs9357347 exerts on AD remains unclear. Therefore, the current study was to test associations of rs9357347 with AD-related cerebrospinal fluid (CSF) and neuroimaging markers in the population from Alzheimer’s Disease Neuroimaging Initiative (ADNI).

## Materials and Methods

### Study Design and Participants

Data used in this study were obtained from the ADNI database led by Principal Investigator Michael W. Weiner, MD, which is a public-private partnership, launched in 2003. The main goal of ADNI has been to test whether clinical and cognitive assessment, positron emission tomography (PET), CSF, serial magnetic resonance imaging (MRI), and other biological markers can be combined to evaluate the progression of mild cognitive impairment (MCI) and early AD. Written informed consent was obtained from all participants or their guardians before test samples being drawn. The institutional review boards of all sites participating in the ADNI provided review and approval of the ADNI data collection protocol. For more details, see www.adni-info.org.

Our ADNI cohort consisted of available baseline and longitudinal AD-related marker samples from all cognitively normal (CN) elders, patients with MCI, and patients with AD dementia. Inclusion or exclusion criteria are described in detail on pages 20–22 of the online ADNI protocol. Briefly, all subjects were aged from 55 to 90 years old, kept contact with their study partners 10 h per week or more, had completed at least six grades of education or had a good employment history, spoke English or Spanish fluently, and were removed of any significant neurological disease other than AD (Petersen et al., 2010). Any history of head trauma or brain lesions, any serious neurological disease other than probable AD, and any psychoactive medication use that could otherwise account for the deterioration in memory and related symptoms must be excluded (Jagust et al., 2009; Weigand et al., 2011).

We used two classification criteria for the 722 ADNI samples to study the association of rs9357347 allele with AD dementia. One is clinical classification (AD, MCI, CN groups) which includes 201 CN older adults, 349 elders with MCI, and 172 elders with AD dementia. And another is pathological classification classifying the subjects into two groups according to previously established cutoff. It has been shown that individuals with CSF Aβ_1-42_ levels less than 192 pg/ml in the ADNI cohort have evidence of Aβ_1-42_ deposition in the brain, as detected by PET-PIB (Fagan et al., 2007; Mattsson et al., 2009). Individuals with CSF Aβ_1-42_ levels below these thresholds (CSF Aβ_1-42_ ≤ 192 pg/ml) could be classified as Aβ-positive (Aβ+), otherwise (CSF Aβ_1-42_ > 192 pg/ml) they will be classified as Aβ-negative (Aβ-) (Olsson et al., 2005; Jack et al., 2008; Shaw et al., 2009). Thus, 259 Aβ+ elders and 117 Aβ- elders were selected from ADNI database.

### CSF Biomarkers

The CSF data used in this study were obtained from ADNI dataset. CSF sampled by lumbar puncture was gathered into collection tubes, and then transferred to polypropylene conveying tubes. After collection, the samples were frozen on dry ice within 60 min and transported immediately to the ADNI Biomarker Core laboratory at the University of Pennsylvania Medical Center. Preparation of aliquots (0.5 ml) from these samples was done after unfreezing (60 min) at room temperature and gentle mixing (Weigand et al., 2011). The aliquots were stored in bar code-labeled polypropylene vials at -80°C (Shaw et al., 2009). The multiplex platform (xMAP; Luminex Corporation) with a kit (INNO-BIA AlzBio3; Fujirebio Europe) was used for simultaneous measurement of the CSF protein biomarkers such as Aβ_1-42_, total tau (T-tau), and tau phosphorylated at threonine 181 (P-tau 181). More details for CSF acquisition and measurement have been reported previously (Olsson et al., 2005).

### Brain Structures on MRI

The volumes of brain structures in MRI used in our study were from the UCSF data in ADNI dataset^1^. Firstly, we downloaded preprocessed MRI data from Laboratory of Neuroimaging (LONI) IDA^2^ with 1.5T or 3T data available. Then cerebral image analysis and segmentation were performed with the Free Surfer version 5.1 including corrected for motion, averaged, normalized for intensity. The technical details of these procedures are described in prior publications (Jack et al., 2008). Here, we selected the most associated brain regions with AD, such as hippocampus, ventricle and middle temporal as our regions of interest (ROI).

### Glucose Metabolism on FDG-PET

The information related to glucose metabolism was from the UC Berkeley and Lawrence Berkeley National Laboratory (Landau et al., 2011). The brief routine processes were as follows. First of all, PET data were downloaded from LONI website^3^. Then the mean counts from the meta ROIs (left and right inferior/middle temporal gyrus, left and right angular gyri, and bilateral posterior cingulate gyrus) for each subject’s FDG scans at each time point were extracted and the intensity values were calculated with SPM5 subroutines. Finally, mean FDG uptake was extracted for each of the five ROIs and normalized by dividing it by pons/vermis reference region mean (Landau et al., 2011). Total FDG uptake was calculated as a mean of the five individual Meta ROIs ^4^.

### Statistical Analysis

Associations between diagnosis and demographic, clinical factors were tested at baseline. We examined differences in continuous variables (education years, age, volume, etc.) using Kruskal–Wallis test. We tested differences in Categorical data (gender, APOE 𝜀4 status) using chi-square test. The correlations between rs9357347 and various endophenotypes (CSF proteins, MRI and FDG-PET) were estimated using linear regression models at baseline. Furthermore, association between rs9357347 and the above phenotypes in the longitudinal study were tested using linear mixed-effects models. Of note, we chosen the 4-year follow-up data for all three existing genotypes to make analysis. We used mixed linear models that specified a random subject-specific intercept and a random subject-specific slope. All outcome variables in linear regression models and linear mixed-effects models were standardized to *z* scores to facilitate comparisons between genotypes. Difference with a *P*-value < 0.05 was considered to be statistically significant. All regression analyses were corrected for age, gender, APOE 𝜀4 genotype and educational level, and the regression analysis of brain structure volume was also corrected for intracranial volume. All statistical analyses were performed by R3.2.0^5^.

## Results

### Baseline Characteristics of Study Participants

The study population was composed of 201 CN elders, 349 elders with MCI, and 172 elders with AD. Demographic and clinical characteristics at the baseline were summarized in the Table 1. As expected, the frequency of the APOE 𝜀4 allele in AD group was significantly higher than those in MCI and CN group. Among the three groups, the diagnosis was found to be correlated with gender, education level, volume of brain structure, and FDG. The diagnosis did not differ by age. Individuals in AD and MCI cohorts exhibited typical CSF biomarker phenotype of AD with elevated mean levels of T-tau and P-tau181 and lower level of Aβ_1-42_.

**Table 1:** The characteristics of the subjects in clinical group at baseline.

Characteristics	CN (N = 201)	MCI (N = 349)	AD (N = 172)	P value
Age (years)	75.80 (4.84)	74.76 (7.34)	75.42 (7.40)	0.56
Gender (male/female)	107/94	229/120	91/81	<0.01
Education (years)	16.08 (2.82)	15.64 (3.08)	14.64 (3.17)	<0.01
ApoE 𝜀4 (0/1/2)	147/50/4	156/151/42	57/84/31	<0.01
Aβ (pg/ml)	206.33 (54.35)	162.86 (52.31)	143.39 (38.09)	<0.01
T-tau (pg/ml)	69.85 (30.25)	103.04 (58.31)	121.07 (56.80)	<0.01
P-tau (pg/ml)	25.39 (14.80)	36.17 (19.26)	41.92 (20.27)	<0.01
FDG	1.29 (0.12)	1.20 (0.12)	1.09 (0.13)	<0.01
Hippocampus (mm^3^)	7279.21 (888.23)	6381.58 (1074.03)	5596.41 (1063.70)	<0.01
Middle temporal (mm^3^)	19921.81 (2790.99)	18589.17 (2912.60)	16827.31 (3131.44)	<0.01
Ventrical (mm^3^)	35088.02 (19446.31)	44700.22 (23944.76)	49504.77 (25596.41)	<0.01

*CN, cognition normal; MCI, mild cognitive impairment; AD, Alzheimer’s disease; Aβ, β-amyloid; T-tau, total tau; P-tau, tau phosphorylated at threonine; FDG, F18-fluorodeoxyglucose. *N* indicates the number of subjects from each group with at least 1 follow-up measure available. Data are mean (standard error). *p* values for continuous variables are from Kruskal–Wallis test. *p* values for categorical data are from chi-square test.*

### Impacts of rs9357347 on CSF Markers

**FIGURE 1:**
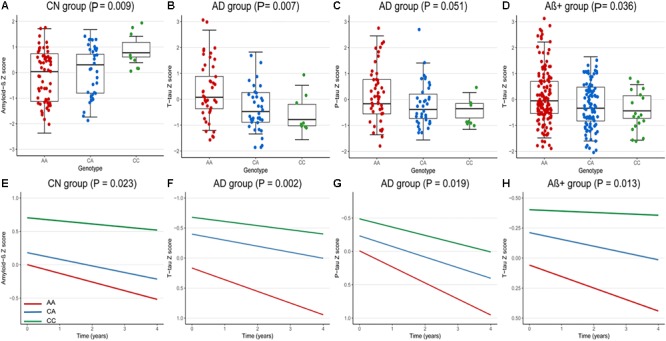
The correlation between rs9357347 and CSF markers. **(A–D)** The relation between CSF protein and rs9357347 allele at baseline. **(A)** The statistical relation between CSF Aβ_1-42_ and rs9357347 allele in CN group; **(B)** rs9357347 was associated with the level of T-tau in AD group; **(C)** rs9357347 was closely associated with P-tau in AD group; **(D)** rs9357347 was associated with the level of T-tau in Aβ-abnormal (CSF Aβ_1-42_ ≤ 192 ng/L) group; **(E–H)** The relation between CSF protein and rs9357347 allele longitudinally (4 years’ follow-up). **(E)** The statistical relation between CSF Aβ_1-42_ and rs9357347 allele in CN group; **(F)** rs9357347 was associated with the level of T-tau in AD group; **(G)** rs9357347 was associated with P-tau 181 in AD group; **(H)** rs9357347 was associated with the level of T-tau in Aβ-abnormal group; The model at baseline was multiple linear regression model adjusted for age, gender, educational level, and APOE 𝜀4 genotype. The model in longitudinal study was multiple mixed-effects model adjusted for age, gender, educational level, and APOE𝜀4 genotype. Aβ+, Aβ-abnormal; Aβ–, Aβ-normal.

At baseline, we analyzed the correlations between rs9357347 and concentration of CSF proteins in three clinical groups (CN, MCI, and AD) and two pathological groups (Aβ+, Aβ-). In CN group, the significant correlation was seen with CSF Aβ_1-42_ (β = 0.36, *P* = 0.009) (Figure 1A). In AD group, rs9357347 was found to be correlated with CSF T-tau (β = -0.44, *P* = 0.007) (Figure 1B). Meanwhile, we discovered possible relation between rs9357347 and CSF P-tau 181 in AD group (β = -0.31, *P* = 0.051) (Figure 1C). As for the pathological group, we also found the obvious association between rs9357347 and CSF T-tau in Aβ+ group (β = -0.20, *P* = 0.036) (Figure 1D).

In longitudinal study, the findings were similar to the results at baseline after adjusting for age, gender, education level, APOE 𝜀4. In CN group, CSF Aβ_1-42_ was found to be correlated with the locus (β = 0.33, *P* = 0.023) (Figure 1E). Furthermore, in AD group, rs9357347 was found to exert a strong effect on CSF T-tau (β = -0.47, *P* = 0.002) (Figure 1F) and P-tau 181 (β = -0.33, *P* = 0.019) (Figure 1G), while we failed to discover similar correlation in MCI and CN groups. And when the samples were stratified by pathological status, the C allele of rs9357347 was found to decrease the CSF T-tau (β = -0.24, *P* = 0.013) (Figure 1H) in Aβ+ group.

### Impacts of rs9357347 on MRI Structure and Glucose Metabolism

Association of rs9357347 with AD-related neuroimaging measures and FDG-PET imaging were investigated at baseline and longitudinally. Neither AD-related brain structures (right and left hippocampus, middle temporal gyrus and ventricle) nor cerebral metabolism rate of glucose (CMRgl) on FDG-PET imaging was found to have obvious association with rs9357347.

## Discussion

In this study, we aimed to characterize the pathogenic effect rs9357347 exerted on AD based on the premise that rs9357347, a candidate regulatory variant at the *TREM* gene cluster, associates with decreased AD risk and increased *TREML1* and *TREM2* brain gene expression (Carrasquillo et al., 2017). We found statistically significant associations with both Aβ_1-42_ and tau in CSF (at baseline and longitudinally). Specifically, we reported that rs9357347 was mainly associated with CSF Aβ_1-42_ in CN participants; in AD participants, the mutation was detected to be negatively associated with CSF T-tau, which is consistent with the result in Aβ+ group; the association between the locus and CSF P-tau 181 was also found in AD group longitudinally. To sum up, our results were in line with the hypothetical model that relates disease stage to AD biomarkers, in which CSF Aβ_1-42_ biomarkers become abnormal first and then the neurodegenerative biomarkers (Hardy and Selkoe, 2002; Dennis and Selkoe, 2016). This suggests that there is an association of rs9357347 with brain amyloidosis and tau pathology of AD. This finding may supply clues to the protective role of rs9357347 against AD risk.

Previous studies have proposed a mechanism by which genetic variation in *TREM2* alters amyloid pathology. First of all, TREM2 is a cell surface receptor of the immunoglobulin superfamily, which is expressed nearly exclusively on microglia within the central nervous system (CNS) (Zheng et al., 2016). Second, in CNS, TREM2 signaling is intimately linked with the adapter protein, DAP12 (also known as TYRO protein tyrosine kinase binding protein, TYROBP) (Li and Zhang, 2018). TREM2 deficiency is associated with decreased bacterial clearance and increased pro-inflammatory cytokine production, thus suggesting the anti-inflammatory and protective functions of TREM2 (Zheng et al., 2016). The number of myeloid cells around amyloid plaques was decreased in TREM2 hemizygous mutations showed that a loss of TREM2 function results in reduced Aβ_1-42_ uptake (Yuan et al., 2016). Further, a previous study found that microglia became more active to remove amyloid plaques after *TREM2* overexpression, and the density of amyloid plaques in the brain reduced in middle-aged APP/ PS1 mice (7–8 months old) (Jiang et al., 2014). Condello et al. (2018) postulated that the microglia around the amyloid surface limits fibril outgrowth and plaque-associated toxicity.

Many of studies suggested that *TREM2* participates in AD pathogenesis through intraneuronal deposition of phosphorylated tau besides Aβ_1-42_ deposition and clearance (Jay et al., 2015; Jiang et al., 2015, 2018). Tau pathology in the CNS, current is involved in a series of neurodegenerative disorders including AD (Medina and Avila, 2014). Therefore, regulation of tau pathology may be an impactful approach to delaying the AD development. Mutations in *TREM2* have been suggested to be correlated with tau pathology in AD. Lill et al. (2015) reported that a variant of *TREM2* (rs75932628) significantly increased the accumulation of CSF T-tau in a European population, which suggested that *TREM2*’s role in AD may involve neuronal degeneration. additionally, the overexpression of *TREM2* has been found to significantly enhance hyperphosphorylation of tau proteins (Lill et al., 2015). Consequently, *TREM2* overexpression significantly improves neuronal loss and may play a role in the phosphorylation of tau protein, thereby improving the incidence of AD. Of note, inflammatory stimuli in the brain have also been proved to be associated with accelerating tau phosphorylation (Jiang et al., 2016). Since tau phosphorylation would be accelerated resulting from reduced TREM2 levels suggesting that TREM2 act as an anti-inflammatory factor. In the light of our result, we can hypothesize that the upregulation of *TREM2* driven by the rs9357347-C allele serves as a compensatory response to Aβ_1-42_ and tau and subsequently protects against AD progression.

Currently, TREML1 (also named as TLT-1) has been identified as a myeloid receptor expressed exclusively in the α-granules of megakaryocytes and platelets (Washington et al., 2004). TREML1 was proved to antagonize proinflammatory activation of TREM1 by competing with its ligand (Derive et al., 2012). Besides, soluble TREML1 has also been shown to play an anti-inflammatory role. Meanwhile, *TREML1* levels in the brain have reported to have association with decreased AD risk in humans (Carrasquillo et al., 2017) suggesting that upregulated expression of *TREML1* may be protective against AD. Nevertheless, as we all know, no variant in TREML1 is currently exerted to associated with AD (Derive et al., 2012). For that reason, our results that rs9357347 exerted a significant effect on AD by modulating CSF biomarkers including Aβ_1-42_, T-tau, and P-tau 181 has thrown light on the hypothesis about the mechanism through which TREML1 modifies AD risk, which still needs further investigation.

Our findings were consistent with the results of Carrasquillo et al. (2017) that rs9357347 has functional influence on AD and may indicated the role that rs9357347 played on protection from AD. The study demonstrated that the locus was associated with the level of Aβ and tau. Thus far, it has been identified that both Aβ and tau pathology could lead to neuronal dysfunction and neurodegeneration (Jack et al., 2010; Wang et al., 2015). Many studies suggested that TREM2’s role in AD may involve tau dysfunction and Aβdeposition (Lill et al., 2015; Zheng et al., 2016). All the above evidence, along with our findings, supported that rs9357347 mediated AD risk by modulating the alteration of the amyloid-β pathology and neuronal degeneration biomarkers. Although our results showed evidence that Aβ_1-42_ and tau response were in part mediated through rs9357347, there remained a possibility that tau effects may be also derived from an imbalance between Aβ_1-42_ production and clearance (Hardy and Selkoe, 2002; Selkoe and Hardy, 2016). A detailed analysis of the potential significance of secondary changes is indeed interesting, so future studies are needed to robustly identify the locus for the associations with Aβ_1-42_ and tau pathology.

## Conclusion

In summary, our findings confirmed that the rs9357347^CC^ exerted a protective effect on AD by decreasing the Aβ_1-42_ accumulation and tau burden. These findings further supported the hypothesis that *TREM2* and *TREML1* may modulate amyloid-β pathology and neuronal degeneration to influence the risk of AD.

## Author Contributions

J-QL and C-CT contributed to the conception and design of the study. M-LT and X-NN performed the statistical analysis. M-LT wrote the manuscript. X-PC, J-QL, C-CT, and LT contributed to the manuscript revision, read and approved the submitted version of the manuscript.

## Conflict of Interest Statement

The authors declare that the research was conducted in the absence of any commercial or financial relationships that could be construed as a potential conflict of interest.

## Footnotes

1 https://ida.loni.usc.edu/pages/access/studyData.jsp

2 https://ida.loni.usc.edu

3 http://loni.usc.edu/

4 www.adni-info.org

5 http://www.r-project.org/
